# AFM Imaging Reveals MicroRNA‐132 to be a Positive Regulator of Synaptic Functions

**DOI:** 10.1002/advs.202306630

**Published:** 2024-03-17

**Authors:** Ikbum Park, Hyun Jin Kim, Juyoung Shin, Yu Jin Jung, Donggyu Lee, Ji‐seon Lim, Jong Mok Park, Joon Won Park, Joung‐Hun Kim

**Affiliations:** ^1^ Technical Support Center for Chemical Industry Korea Research Institute of Chemical Technology (KRICT) Ulsan 44412 Republic of Korea; ^2^ Department of Life Sciences Pohang University of Science and Technology (POSTECH) Pohang 37673 Republic of Korea; ^3^ Center for Specialty Chemicals Korea Research Institute of Chemical Technology (KRICT) Ulsan 44412 Republic of Korea; ^4^ Division of Electronics and Information System Daegu Gyeongbuk Institute of Science and Technology (DGIST) Daegu 42988 Republic of Korea; ^5^ Department of Chemistry Pohang University of Science and Technology (POSTECH) Pohang 37673 Republic of Korea; ^6^ Institute of Convergence Science Yonsei University Seoul 03722 Republic of Korea

**Keywords:** atomic force microscopy, dendritic spines, force‐mapping, microRNA, synaptic strengthening

## Abstract

The modification of synaptic and neural connections in adults, including the formation and removal of synapses, depends on activity‐dependent synaptic and structural plasticity. MicroRNAs (miRNAs) play crucial roles in regulating these changes by targeting specific genes and regulating their expression. The fact that somatic and dendritic activity in neurons often occurs asynchronously highlights the need for spatial and dynamic regulation of protein synthesis in specific milieu and cellular loci. MicroRNAs, which can show distinct patterns of enrichment, help to establish the localized distribution of plasticity‐related proteins. The recent study using atomic force microscopy (AFM)‐based nanoscale imaging reveals that the abundance of miRNA(miR)‐134 is inversely correlated with the functional activity of dendritic spine structures. However, the miRNAs that are selectively upregulated in potentiated synapses, and which can thereby support prospective changes in synaptic efficacy, remain largely unknown. Using AFM force imaging, significant increases in miR‐132 in the dendritic regions abutting functionally‐active spines is discovered. This study provides evidence for miR‐132 as a novel positive miRNA regulator residing in dendritic shafts, and also suggests that activity‐dependent miRNAs localized in distinct sub‐compartments of neurons play bi‐directional roles in controlling synaptic transmission and synaptic plasticity.

## Graphical abstract

The atomic force microscopy (AFM)‐based nanoscale imaging is performed to monitor activity‐dependent changes in specific miRNAs in the sub‐compartments of single neurons. This imaging reveals increases in the amounts of miR‐132 in the dendritic regions abutting functionally‐active spines, providing insights into how neurons can modulate internal milieu to changing external stimuli such as synaptic plasticity.

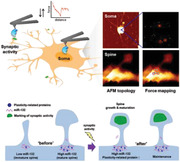

## 1 Introduction

Synaptic plasticity, which involves long‐lasting functional changes in synaptic and neuronal connectivity, underlies learning and memory.^[^ ^1^, ^2^, ^3^ ^]^ For instance, long‐term potentiation (LTP) and long‐term depression (LTD) are further stabilized by structural changes in synaptic components, which require trafficking or synthesis of *de novo* proteins.^[^ ^1^, ^2^ ^]^ Spines, which are small protrusions emanating from the dendritic shafts of mammalian neurons, are major postsynaptic structures in apposition to axonal terminals of excitatory synapses that undergo functional and structural changes.^[^ ^4^, ^5^ ^]^ The synaptic efficacy of individual spines can be modulated in an activity‐dependent manner,^[^ ^6^ ^]^ mainly at activated synapses subject to increased neural activity, and this modulation contributes to formation and expression of specific memory.^[^ ^3^, ^7^ ^]^

MicroRNAs (miRNAs) are single‐stranded RNAs that can bind to mRNAs with complementary sequences, which normally leads to their translational inhibition and/or degradation.^[^ ^8^, ^9^ ^]^ Since a subset of neuronal miRNAs can suppress expression of the target proteins that comprise synapses and regulate synaptic features, they can play pivotal roles in synapse maturation and synaptic plasticity throughout an animal's lifespan.^[^ ^10^, ^11^ ^]^ Consistent with such modulatory roles, it was previously reported that certain miRNAs are enriched in spines,^[^ ^12^, ^13^ ^]^ and that brain‐specific miRNAs can be up‐ and down‐regulated in an activity‐dependent fashion.^[^ ^14^, ^15^, ^16^ ^]^ Given this activity dependency, as well as the intrinsic effects of miRNAs on protein production, miRNAs are likely to control the distribution and abundance of synaptic proteins at select (active) synapses, and thereby dictate synaptic and neuronal plasticity.

Indeed, it was previously shown that brain‐specific miRNA species can fine‐tune and modulate synaptic transmission and plasticity.^[^ ^8^, ^9^ ^]^ Although mounting evidence substantiates the physiological importance of miRNAs in neural circuits, most previous studies point to the repressive (negative) roles of miRNAs in synaptic functions and plasticity: deletion and/or depletion of miRNAs increases synaptic efficacy and promotes synaptic plasticity, whereas their overexpression leads to impairment in synaptic functions and deficits in memory formation.^[^ ^17^ ^]^ The general lack of experimental evidence for promotive (positive) roles of miRNAs is because: 1) miRNAs have solely inhibitory effects on nervous systems, or 2) the empirical methods used in previous investigations did not enable sufficiently‐fine resolving of the sub‐cellular actions of miRNAs to elucidate their positive impacts on individual synapses. In fact, miRNAs have been shown to elicit distinct functions across cellular loci and synaptic structures.^[^ ^18^, ^19^ ^]^ Moreover, activity‐induced biochemical and structural changes are heterogeneous across various neuronal sub‐compartments, and are potentially mediated by miRNAs residing therein, leading to cellular and physiological consequences such as soma‐dendritic asynchronization and site‐specific memory allocation.^[^ ^20^, ^21^ ^]^ The detailed delineation of miRNA functions requires in situ analysis of their cellular outcomes at each neuronal structure, and determination of the associations of physiological outcomes with these synaptic structures, which should provide a better understanding of the functional impacts of miRNAs. However, the distribution of miRNAs within single synapses or neuronal compartments has not been fully elucidated, largely because of the technological limits of conventional miRNA detection methods. For instance, direct visualization and quantitative measurement of miRNAs in each sub‐compartment remains challenging, although attempts have been made to identify the heterogeneous distributions of miRNAs using in situ hybridization‐based fluorophore labeling approaches.^[^ ^22^, ^23^ ^]^

**Figure 1:**
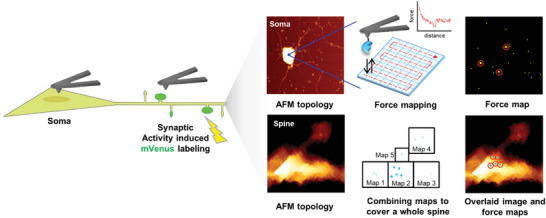
A schematic illustrating miRNA detection with force‐based AFM. To assess activity‐dependent changes in miRNA amounts in sub‐compartments of single neurons, the functional states of dendritic processes and spines are determined using green fluorescence‐tagged ArcMin‐AS. Initially, fluorescence and topology images are captured, followed by categorization of the somas and various types of dendritic spines. In subsequent stages, force maps are generated by applying an HBD‐tethering probe to somas (at three arbitrary positions) and entire dendritic processes. Finally, the spine topology and force maps are superimposed to generate a comprehensive force map, an individual miRNA is represented as a cluster of positive pixels within the adhesion force map.

We recently demonstrated that the precise localization of individual miRNAs can be analyzed using atomic force microscopy (AFM)‐based imaging without the need for amplification. This study revealed that miRNA (miR)‐134 was highly enriched in dendrites and spines with reduced activity, where it would mediate inverse plasticity as a negative regulator.^[^ ^12^, ^24^, ^25^ ^]^ MicroRNA‐132 is an miRNA that shows enrichment in the brain, and its expression is increased with maturation of hippocampal pyramidal and granule cells.^[^ ^26^ ^]^ Interestingly, the enzyme deacetylase sirtuin 1 (SIRT1), which is targeted and suppressed by miR‐132,^[^ ^27^ ^]^ promotes synaptic plasticity and memory formation through the inhibition of miR‐134 expression.^[^ ^25^ ^]^ If miR‐132 is involved in synaptic plasticity and memory formation in this way, miR‐132 could be recruited to active synapses, where it would then play regulatory and instructive roles on localized synaptic plasticity and memory allocation, distinct to or opposing that of miR‐134. In this study, we leveraged the same AFM imaging method to analyze the distribution of miR‐132 at single spine and dendrite levels (**Figure** **1**), in order to examine whether miR‐132 is a positive miRNA that can support synaptic transmission and site‐specific synaptic plasticity.

## 2 Results

AFM offers exceptional lateral resolution (up to nanometers), facilitating the visualization and characterization of individual molecules,^[^ ^28^, ^29^, ^30^ ^]^ which can be employed to monitor molecular interactions under physiological conditions.^[^ ^31^, ^32^, ^33^, ^34^, ^35^, ^36^ ^]^ We previously developed a novel AFM system using a hybrid binding domain (HBD) to detect and quantify individual miRNAs within single cells.^[^ ^12^, ^37^ ^]^ This HBD can specifically bind to RNA/DNA hybrids that do not naturally exist,^[^ ^38^ ^]^ allowing for systematic and multiplex detection of miRNAs. In brief, after fixation and permeabilization of subject neurons, complementary probe DNAs were allowed to hybridize with the target miRNAs (miR‐132 in this study), which engendered the RNA/DNA hybrids that were subsequently captured by HBD. Through this AFM method, we were able to accurately identify individual miR‐132 molecules at specific neuronal regions (namely, dendritic shafts and spines) at a 10‐nm resolution. We also used ArcMin‐AS (labeled with fluorescence signals) as an activity indicator^[^ ^39^ ^]^ to characterize the attributes of miR‐132 within each synaptic structure with different functionality (Figure 4).

### 2.1 miR‐132 Visualization Within Somatic Regions of Neurons

**Figure 2:**
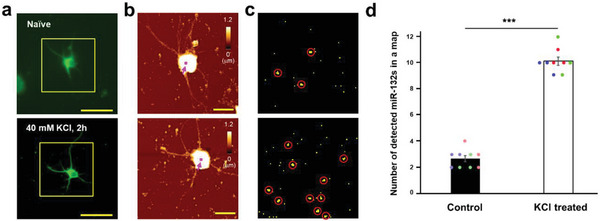
High‐resolution force mapping of membrane depolarization‐induced miR‐132 on neuronal somas. a) Fluorescence images of cultured hippocampal neurons. Neurons are stimulated with saline (top) or 40 mm KCl (bottom) for 2 h and fixed with paraformaldehyde. Neurons are also immunostained with MAP2 for whole‐neuron visualization (green). Scale bars = 30 µm. b) AFM topographic images of boxed regions in (a). Scale bars = 20 µm. c) Representative force maps from naïve (top) and depolarized somas (bottom). An arbitrary position on the soma marked by magenta arrows in (b) is force‐mapped (100 × 100 pixels, 1.0 × 1.0 µm). The pixels where the observed specific adhesion force‐distance curves have a probability of no less than 20% are colored yellow in the force map. The qualifying clusters are outlined with red circles. d) The numbers of miR‐132 molecules in three regions from each neuron (total of three neurons) are compared between naïve and depolarized neurons (^***^ *p* < 0.001, Mann–Whitney test).

To investigate the in situ distribution of miR‐132 within individual neurons, we started to map miR‐132 molecules within somatic regions of hippocampal neurons (**Figure** **2**). After removal of the plasma membrane from cultured hippocampal neurons (DIV14), the comprehensive architecture of individual neurons was elucidated through MAP2 immunostaining. As an initial step, DNA probes complementary to the miR‐132 sequence were applied for hybridization. Then, we obtained high‐resolution force‐based maps at three arbitrary soma positions using the HBD‐tethering AFM probe (HBD‐AFM; Figure 2). The histogram indicated that the most probable adhesion force was 23 pN (Figure S1a,b, Supporting Information). Pixels demonstrating the presence of the force exhibited clustering within the hydrodynamic radius, which confirmed the presence of the targeted miRNA (Figure S1c,d, Supporting Information). The specificity of our analytical approach was corroborated by control experiments with non‐complementary scrambled DNAs and complementary RNAs (Figure S2, Supporting Information). Analogous to earlier observations,^[^ ^12^, ^37^ ^]^ the distribution of miR‐132 appeared uniform across neuronal somas.

Without any treatment, an average of 2.7 miR‐132 molecules were identified in mapped regions (across nine maps obtained from three independent neurons; mapped areas = 1.0 × 1.0 µm). We repeated the same imaging steps after depolarization with a 2‐h potassium chloride (KCl) treatment. This depolarization increased the number of miR‐132 molecules to 9.8 (Figure 2). Interestingly, the distributions and average numbers of miR‐132 molecules observed in somatic regions were comparable with those of miR‐134 before and after depolarization.^[^ ^12^ ^]^

### 2.2 miR‐132 Visualization in Dendrites and Spines

**Figure 3:**
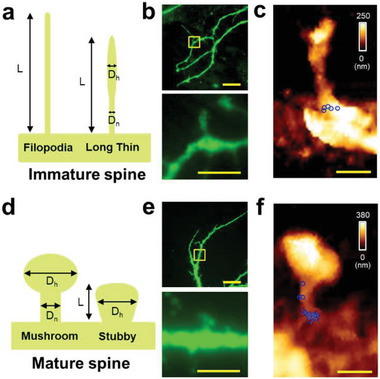
Spine classification and visualization of miR‐132 at individual spines. a) A schematic for spine classification parameters. Spines are classified as immature when protrusion structures match the following criteria: protrusion length (L) of 0.75–2 µm (protrusions over 2 µm were classified as filopodia), and head diameter (*D*_h_) < 1. b) Representative fluorescence images (DIV14): the lower panel shows the boxed area in the upper panel at a higher magnification (MAP2, green). c) AFM topographic image (3.0 × 5.0 µm) of the boxed area in (b) and the force map of individual miR‐132/DNA hybrids are overlaid to obtain the final map. Blue circles indicate miR‐132 molecules whose sizes match the hydrodynamic diameter observed. d) A schematic for the spine classification parameters. Spines are classified as mature spines when protrusion structures matched the following criteria: a ratio of *D*_h_ to neck diameter (*D*_n_) of ≥ 2.5, *D*_h_ > 1, or L ≤ 0.75 µm. e) Representative fluorescence image (DIV14): the lower panel shows the boxed area in the upper panel at a higher magnification (MAP2, green). f) AFM topographic image (3.0 × 5.0 µm) of the boxed area in (e). Sky‐blue pixels represent those pixels in which the specific unbinding event was observed in more than two of five measurements (pixel size, 10 nm), while blue circles indicate miR‐132 molecules whose sizes match the hydrodynamic diameter observed at high resolution. Scale bars = 20 µm (panel b and e, upper panel), 5.0 µm (panel b and e, bottom panel), or 1.0 µm (panel c and f).

We also mapped miR‐132 throughout the dendrites and spines. Prior to force mapping, fluorescence images were captured to delineate surface‐scanning cellular morphology, which enabled the classification of spines into two classes: a mature class with stubby‐shaped and mushroom‐shaped spines, and an immature class with thin spines (**Figure** **3**a,d). In the subsequent phase, force maps of selected spine regions were acquired using HBD‐AFM probes. Typically, more than five force maps were collected, which allowed coverage of the whole spines. These obtained maps were then stitched together to encompass the entire spine region, and the force and morphology maps were then overlaid (Figure 1). Unlike their even distribution within somas (Figure 2), miR‐132 molecules exhibited pronounced localization within specific areas of dendrites and somas. Specifically, miR‐132 molecules were predominantly identified within a confined area measuring 700 nm (horizontal direction) by 200 nm (longitudinal direction), which was positioned around the dendritic shafts aligned beneath corresponding spines. However, miR‐132 molecules were rarely observed in the necks and heads of spines (Figure 3; Figure S3, Supporting Information). Notably, the number of miR‐132 molecules in each region varied, depending on the type of the nearby spines that miR‐132 was located below: the mature spine class exhibited 11.2 ± 0.49 miR‐132 molecules, whereas the immature spine class showed only 5.00 ± 0.32 miR‐132 molecules (Figure 3; Figure S3 and Table S1, Supporting Information). Since miR‐132 molecules were predominantly localized in the dendritic shaft at the base of specific spines, the abundance was most likely to be governed by the functionality of the nearby spines, which could be assessed by their morphology.

### 2.3 Abundance of miR‐132 was Controlled by Activity of Nearby Spines

Synaptic maturation and the ensuing increase in synaptic efficacy supported synaptic transmission and synaptic plasticity, which involved protein production at specific synaptic sites.^[^ ^40^, ^41^, ^42^ ^]^ Since higher miR‐132 levels appeared primarily near the mature dendritic spine types, we sought to examine whether spontaneous local activity of individual synapses could affect the abundance of miR‐132 or not. To address this notion, we employed an adeno‐associated virus that encoded ArcMin‐AS as an activity indicator containing the mVenus cassette for visualization and the dendritic targeting element (DTE) of Arc mRNA as an activity actuator, which ensured selective translation in active sites without interaction with the postsynaptic density (PDZ).^[^ ^39^ ^]^

**Figure 4:**
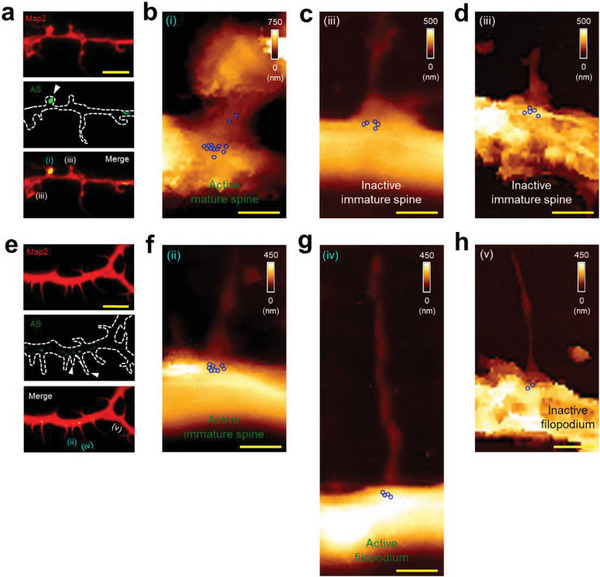
Mapping of individual miR‐132 molecules in filopodia and dendritic spines labeled with an activity marker ArcMin‐AS. a,e) Fluorescence images of filopodia and dendritic spines (top, MAP2, red; middle, AS, ArcMin‐AS, green; bottom, the merged images). Each arrowhead indicates active spines and filopodia (Scale bars = 10 µm). Analyzed dendritic protrusions are assessed as follows: i) Active mature spines, ii) active immature spines, iii) inactive immature spines, iv) active filopodium, and v) inactive filopodium. Scale bars = 5 µm. b–d,f–h) AFM topographic images and force maps obtained from indicated protrusions (i–v) in each panel, respectively, at the same magnification (3.0 × 5.0 µm or 3.0 × 8.0 µm; Scale bar = 1 µm).

**Figure 5:**
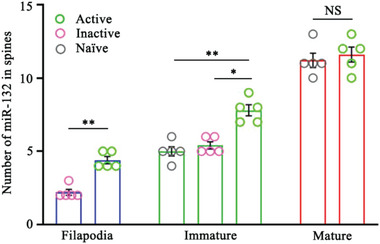
Comparison of miR‐132 molecules between various classes of dendritic protrusions and in accordance with the activity defined by ArcMin‐AS in cultured hippocampal neurons (DIV14). Five protrusions are examined for each case. For raw data, blank gray circles represent naïve dendritic protrusions, blank green circles represent active dendritic protrusions, and blank magenta circles represent inactive protrusions. Error bars indicate standard error of the mean (s.e.m), (filopodia, inactive versus active, *p* = 0.0079; immature, naïve versus active, *p* = 0.0069, inactive versus active comparison, *p* = 0.0416; no significant difference detected in mature spines, one‐way ANOVA followed by Tukey's *post hoc* tests).

Indeed, the transduction of ArcMin‐AS allowed us to readily distinguish both active dendritic spines and filopodia from non‐functional ones (**Figure** **4**a,e; Figure S4a,c,e,g,i,k,m, Supporting Information). Along with the dendrite‐wide morphology marker MAP2, fluorescence signals from ArcMin‐AS revealed where active synaptic protrusions were placed within dendritic processes. We were also able to use HBD‐AFM to quantify individual miR‐132 molecules throughout the same dendrites and spines. A mean of 11.60 ± 0.51 miR‐132 molecules were manifest in the dendrites next to ArcMin‐AS‐expressing (active) mature spines (Figure 4b; Figure S4b,d,l,n; (i)‐labeled panels and Table S1, Supporting Information), a value comparable to the average numbers of miR‐132 molecules next to mature spines delineated by their morphology alone (Figures 3d–f and 5; Figure S3c,d and Table S1, Supporting Information). Interestingly, thin filopodia and immature forms of spines also exhibited mVenus fluorescence, albeit at a considerably lower frequency than the mature class of spines. In these active filopodia and immature spines, the mean numbers of miR‐132 molecules were 4.40 ± 0.24 and 7.80 ± 0.37, respectively (Figures 4f,g and 5, Figure S4b,d,f,h,j,l; (ii) or (iv)‐labeled panels and Table S1, Supporting Information). By contrast, filopodia and immature spines lacking mVenus exhibited mean values of 2.20 ± 0.20 and 5.40 ± 0.24 miR‐132 molecules, respectively (Figure 4c,d,h and 5, Figure S4b,d,f,h; (iii) or (v)‐labeled panels and Table S1, Supporting Information).

The abundance of miR‐132 seemed to be elevated in proportion to the functionality of individual protrusions, except in the mature class of spines (**Figure** **5**). Thus, the abundance of miR‐132 seemed to correlate with the maturation stage of the dendritic protrusions. Even immature protrusion types exhibited certain levels of functionality, as elucidated through ArcMin‐AS, which could be reaffirmed by the abundance of miR‐132. This implies that miR‐132 is a reliable indicator of the functional maturation of neighboring dendritic protrusions that are able to contribute to synaptic transmission and memory formation.

## 3 Discussion

Accumulating evidence indicates the involvement of miRNAs in synaptic transmission and synaptic plasticity.^[^ ^19^, ^40^, ^43^ ^]^ Given the distinct functional impacts of miRNAs within sub‐compartments of neurons, the spatial and functional characterization of individual miRNAs should be imperative for better understanding of their physiological roles. In this study, we employed an HBD‐AFM method enabling nanoscale imaging to unveil the spatial distribution of miRNAs without a further amplification process. Indeed, our AFM imaging revealed that activity‐dependent miR‐132 in dendritic regions reflected the activity states of nearby spines.

### 3.1 Spatial Distribution and Functionality of Activity‐Dependent miRNAs

MicroRNA‐132 was induced in an activity‐dependent fashion, with its expression in somas being greatly increased by KCl‐mediated depolarization (Figure 2). While the activity‐dependent miRNAs miR‐132 and miR‐134 were uniformly distributed throughout the somas,^[^ ^12^ ^]^ both miRNAs were primarily localized to specific dendritic regions, particularly beneath the spine neck areas. The similar distributions of miR‐132 and miR‐134 that were observed reflect their operational collaboration, with miRNA‐mediated bi‐directional representation of neuronal activity and functional interactions between miR‐132 and miR‐134 for synaptic transmission and prospective plasticity.

Synaptic strength should be dynamically coordinated in an activity‐dependent manner^[^ ^6^ ^]^ mainly at activated synapses. Localized and transient synaptic changes should be established and maintained by the production and trafficking of plasticity‐related products (PRPs), as previously suggested in the “synaptic tagging” hypothesis.^[^ ^41^, ^42^, ^44^ ^]^ Although the molecular identity of PRPs remains largely unknown, synaptic modifications should take place through the recruitment of PRPs^[^ ^45^ ^]^ to active synapses to support synaptic plasticity. MicroRNA‐132 is one of the miRNAs showing enrichment in the brain, and its expression increases throughout the maturation of hippocampal pyramidal and granule cells.^[^ ^26^ ^]^ Thus, miR‐132 promotes the synthesis and trafficking of PRPs to cognate synapses for synaptic plasticity when induced by localized cellular activity.

### 3.2 Coordinated Regulation of miRNAs for Diverse States of Individual Synapses

We found that miR‐132 displayed distinct anatomical distribution in the dendritic protrusions, in a similar manner to miR‐134. The identical dendritic localization of these miRNAs suggests that the bottom of the spine neck is a main area where activity‐dependent miRNAs play their roles in synaptic transmission and plasticity. Since dendritic spine necks are enriched with polysomes,^[^ ^46^ ^]^ which enable rapid protein translation, both positively‐ and negatively‐regulated miRNAs would support the local protein synthesis essential for synaptic transmission and plasticity^[^ ^2^, ^47^, ^48^, ^49^, ^50^ ^]^ by controlling the translation of various PRP mRNAs in dendrites and spines.

**Figure 6:**
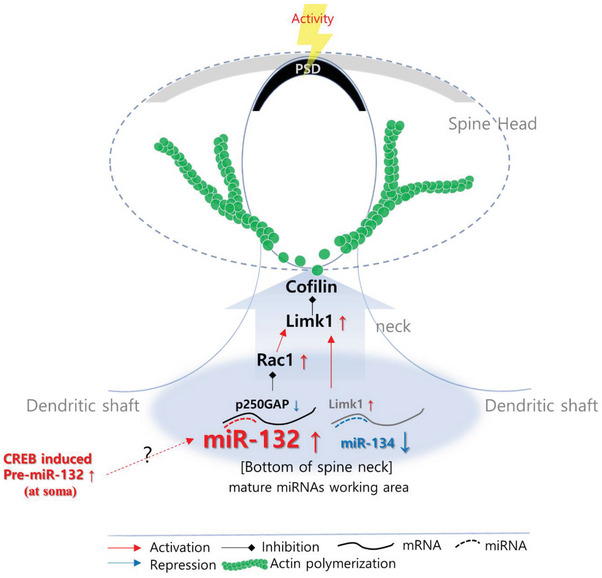
A regulatory model for miRNA‐mediated spine maturation. Activity‐dependent miRNA‐132 and ‐134 are distinctly localized together at the base of spine necks, exhibiting bidirectional regulation of their levels in response to activity. miR‐132‐mediated suppression of p250GAP results in the disinhibition of Rac1 and subsequent activation of Limk1. This process is co‐regulated by the activity‐dependent reduction in miR‐134. Consequently, Limk1 activation leads to cofilin inactivation through phosphorylation, promoting actin polymerization. Nonetheless, the presence of modulators that mediate the bidirectional activity‐dependent regulation of miRNAs remains uncertain.

Even elevation of somatic miR‐132 following KCl‐mediated depolarization seemed to be triggered by CREB activation within 2 h. However, miR‐132 abundance was locally controlled by the spontaneous activity of nearby spines. We attribute this differential distribution of miR‐132 between somas and dendrites to spatial differences in the sensitivity of transcription to activity, or alternative induction mechanisms. Thus, while miR‐132 and miR‐134 occupied similar neuron loci but exhibited opposite responses to synaptic activity, it remained unclear whether they could be simultaneously or sequentially induced in dendrites. Besides the possible roles in production and trafficking of PRPs in dendrites abutting active spines, localized induction of miR‐132 could inhibit p250GAP and consequently activate LIM domain kinase 1 (LimK1) through disinhibition of Rac1, which would result in structural alteration of individual synaptic structures, most likely through the control of cofilin‐mediated actin polymerization (**Figure** **6**).

### 3.3 An Activity Indicator and Predictor of Synaptic Transmission and Plasticity

MicroRNA‐132 was previously demonstrated to be induced by activation of CREB.^[^ ^51^, ^52^, ^53^ ^]^ Consistent with this notion, the abundance of miR‐132 increased in proportion to the cellular activity of nearby dendritic protrusions, as assessed by the Arc‐mediated site‐specific activity marker (Figure 4). Thus, miR‐132 seemed to have the potential to serve as a reliable proxy for cellular activity occurring in parallel with CREB activation. In fact, the distribution profiles of miR‐132 showed consistent increments with low variation, high localization beneath the neck of spines, and selectivity to active sites, further arguing for the efficacy of miR‐132 as an activity indicator at single spine and dendrite levels. Importantly, the abundance of miR‐132 molecules increased within dendritic regions next to both immature and mature active protrusions, which supports the possibility that miR‐132 would be an even more reliable marker of the activity of synaptic structures than their morphology alone. The presence of and enrichment with miR‐132 would predispose individual dendritic protrusions to synaptic competence, and might predict their plasticity by regulating PRP synthesis. Further investigations involving phenotypic comparisons and physiological analyses between miR‐132 and conventional activity markers derived from immediate early genes (IEGs) are required for future usage.

In summary, the AFM imaging performed in this study revealed that miR‐132 increased in an activity‐dependent manner, in stark contrast to miR‐134. Indeed, subsequent in situ evidence indicated that miR‐132 was the first miRNA residing in the necks of spines to exert affirmative roles on synaptic activity and subsequent synaptic plasticity. It was reported that dysregulation of miR‐132 triggered anxiety‐related behaviors and contributed to onset of psychiatric diseases such as obsessive‐compulsive disorder,^[^ ^40^, ^54^ ^]^ which indicates the merit of further studies delving into the actions of miR‐132 in physiological and pathological conditions. Therefore, besides proposing miR‐132 as a positive miRNA regulator, we provide insights into the potential mutual contributions of miR‐132 and miR‐134 to activity‐dependent synaptic and structural plasticity.

## 4 Experimental Section

Methods and any associated references are available in the Supporting Information.

## Conflict of Interest

The authors declare no conflict of interest.

## Author Contributions

I.P. and H.J.K. contributed equally to this work. I.P., J.W.P., and J.‐H.K. conceived the project. I.P. and J.‐H.K. designed and coordinated the experiments. I.P. designed the experimental protocol and performed experiments with AFM. Y.J.J., D.L., J.L., and J.M.P. provided AFM experimental support and discussed AFM results. H.J.K. prepared neuronal cells and constructed ArcMin‐AS, supported with S.J. All the authors discussed results. I.P., J.‐H.K., and H.J.K. wrote the manuscript. All authors read and approved the final manuscript.

## Supporting information

Supporting Information

## Data Availability

The data that support the findings of this study are available from the corresponding author upon reasonable request.
