# Supplementary material for: AFM Imaging Reveals MicroRNA‐132 to be a Positive Regulator of Synaptic Functions
Source: Adv Sci (Weinh). 2024 Mar 17;11(17):2306630. doi: 10.1002/advs.202306630 (PMC11077659; doi:10.1002/advs.202306630)
Supplement: Supplementary file 1 — Supporting Information [file ADVS-11-2306630-s001.pdf]

ADVANCED SCIENCE
Open Access

Supporting Information

for *Adv. Sci.*, DOI 10.1002/advs.202306630

AFM Imaging Reveals MicroRNA-132 to be a Positive Regulator of Synaptic Functions

Ikbum Park, Hyun Jin Kim, Juyoung Shin, Yu Jin Jung, Donggyu Lee, Ji-seon Lim, Jong Mok Park, Joon Won Park and Joung-Hun Kim**

Supporting Information

AFM Imaging Reveals MicroRNA-132 to be a Positive Regulator of Synaptic Functions

Ikbum Park, Hyun Jin Kim, Juyoung Shin, Yu Jin Jung, Donggyu Lee, Ji-seon Lim, Jong Mok Park, Joon Won Park, and Joung-Hun Kim**

Ikbum Park, Jong Mok Park
Technical Support Center for Chemical Industry, Korea Research Institute of Chemical Technology (KRICT), Ulsan 44412, Republic of Korea
E-mail: ibpark@krict.re.kr

Joung-Hun Kim, Hyun Jin Kim, Juyoung Shin
Department of Life Sciences, Pohang University of Science and Technology (POSTECH), Pohang 37673, Republic of Korea
E-mail: joungkim@postech.ac.kr

Joung-Hun Kim
Institute of Convergence Science, Yonsei University, Seoul 03722, Republic of Korea

Yu Jin Jung
Center for Specialty Chemicals, Korea Research Institute of Chemical Technology (KRICT), Ulsan 44412, Republic of Korea

Ji-seon Lim, Joon Won Park
Department of Chemistry, Pohang University of Science and Technology (POSTECH), Pohang 37673, Republic of Korea

Donggyu Lee
Division of Electronics and Information System, Daegu Gyeongbuk Institute of Science and Technology (DGIST), Daegu 42988, Republic of Korea

1. Supplementary Methods

1.1. Functionalization of HBD on AFM probes.

We coated AFM probes (MSNL, Bruker, Santa Barbara, CA) with 27-acid dendrons (custom synthesis, VRND NanobioOrganics, India) and generated *N*-hydroxysuccinimide groups on the apex of the dendrons using disuccinimidyl carbonate (Sigma-Aldrich) as described previously.[1] We then immersed NHS-modified AFM probes in reaction with 4-maleimidobutyric acid *N*-hydroxysuccinimide ester (GMBS) (10 mM) and glutathione (GSH) (10 mM). The GSH-immobilized probes were placed in PBS containing GST-fused HBD (200 nM) at room temperature for 2 h and were rinsed with PBST (0.05% Tween 20 (v/v)) and deionized water. The prepared tips were stored in PBS at 4 °C and used within a week.

1.2. Neuron culture.

C57BL/6 mouse hippocampal neurons were dissected from postnatal day one pups and cultured on poly-L-lysine (Sigma)-coated rectangular cover glass (24 mm × 60 mm) with 1 cm height of teflon wall. Neurons were plated in neurobasal medium (Invitrogen) supplemented with 2.0% B27 (Invitrogen), 1.0% GlutaMAX™ (Invitrogen), and 2.0% fetal bovine serum (FBS, Welgene), and 1.0% penicillin/streptomycin (Invitrogen) (v/v) in a humidified 5% CO_2/95% O_2 incubator at 37 °C. After 6–8 h, the medium was replaced with a serum-free conditioned neurobasal medium.

1.3. Activity indication by ArcMin-AS.

To visualize the local activities, we used ArcMin-AS.[2] The construct vector was kindly provided by Dr. Haruo Kasai (Tokyo Univ., Japan). We packed the construct into AAV by following the established protocol.[3] We bath-applied prepared virus AAV2-SARE-ArcMin-PSDΔ1.2-mVenus-PaRac1-SV40 (GC titer: 6.812×10^{12}; 0.5 μl / 2 ml / each chamber) into DIV 11 of cultured neurons and three days later, at DIV14, we fixed the cultured neurons. To trace single neurons, we immuno labeled MAP2 with Alexa-568 for counterstaining mVenus expressing areas of neuronal dendrites and spines using a fluorescence microscope. We used this construct only for the activity indicator.

1.4. Hybridization of probe DNA on fixed neuronal cells.

To avoid RNase contamination, experimental tools and glassware were treated with RNaseZap (Ambion) and ethanol, and deionized water was treated with diethylpyrocarbonate (DEPC, 0.05% (v/v)) for 20 h, and subsequently autoclaved. At DIV14, neurons were fixed with 4.0%

paraformaldehyde in PBS (v/v) at 4 °C for 15 min, and the plasma membrane was permeabilized using 0.20% Triton X-100 in PBS (v/v) at room temperature for 10 min. The fixed and permeabilized neurons were incubated in a hybridization buffer (2× SSPE buffer (pH 7.4) containing 7.0 mM SDS) containing a probe oligonucleotide (20 μM) at 34 °C for 12 h. The cells were washed with hybridization buffer, 2× SSC buffer, and 0.2× SSC buffer sequentially at 60 °C, stirring for 15 min.

The neurons were further processed for immunostaining. The neurons were blocked with 10 % normal goat serum (Gibco) (in PBS (v/v)) at room temperature for 1 h and labeled with an antibody specific to MAP2A (1:500 dilution, MAB378, Millipore) at 4 °C for 1 day. A goat antibody to mouse was used as a secondary antibody (1:500 dilution; for soma investigation: Alexa 488-conjugated, A-11001, Invitrogen; for dendritic spines: Alexa 568-conjugated, A-11004, Invitrogen) at room temperature for 90 min. The prepared samples were immediately examined with AFM.

1.5. AFM experiment and data analysis.

The AFM image and map miRNAs were carried out using a NanoWizard III and IV AFM (Bruker, Germany) equipped with a fluorescence microscope (Axiovert 200, Zeiss, Germany). The AFM cantilever was calibrated with a built-in program based on the thermal fluctuation method (spring constant 0.010-0.020 $N \cdot m^{-1}$). The AFM probe was placed on a neuronal soma or dendritic spines, and the morphology was imaged in QI mode before the force mapping. We recorded adhesion force maps (100 × 100 pixels, 1.0 × 1.0 μm^2) by collecting five force-distance (F-D) curves on each pixel with an approach/retraction speed of 2.0 $\mu m\ s^{-1}$ and z-length of 500 nm. For the approach, the maximum applied force was 80 - 100 pN to minimize mechanical damage to the AFM probe. To observe the hydrodynamic radius of a miR-132/DNA hybrid, a pixel size of 4.0 nm was employed. The collected F-D curves were analyzed using the JPK data processing program and treated as described previously [1]. Considering the observed hydrodynamic diameter (ca. 28 nm), we regarded a cluster of three connected pixels as a qualified one. The most probable values on the force and distance histograms were calculated by Gaussian fitting (OriginPro 8). All statistical analyses comparing miR-132 levels in each group or spine class were conducted with the Prism (9.3.1-Ver.,GraphPad) program.

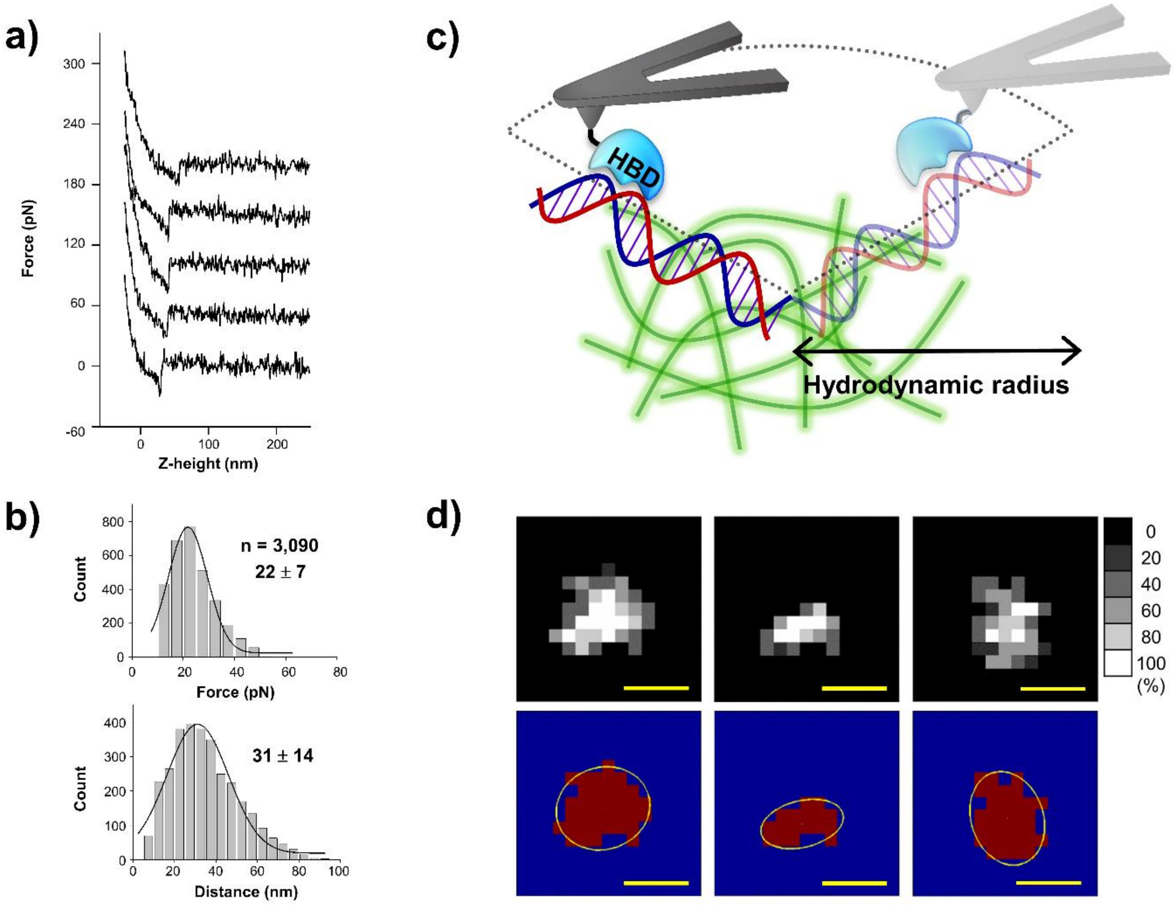

Figure S1. Observation of a cluster of positive pixels made possible by the hydrodynamic distance of HBD and the miR-132/DNA hybrid. a) Unbinding events for the adhesion between HBD and the miR-132/DNA hybrid in soma and dendritic spines. b) Histograms of the adhesion force and distance for the unbinding events. n indicates the number of curves analyzed to generate the histogram. The most probable values are given as the mean ± s.e.m. from the Gaussian fit. c) A scheme for Brownian motion of the HBD and miRNA/DNA hybrid, and their binding at various positions in dendritic spines. d) The upper panel images are created from high-resolution force maps showing an isolated cluster (pixel size 4.0 nm). The maps are acquired on fixed dendritic spines. The lower panel images show a fitted ellipse (yellow circle) used to calculate the radius of each cluster in the upper panel force maps. Scale bars = 20 nm.

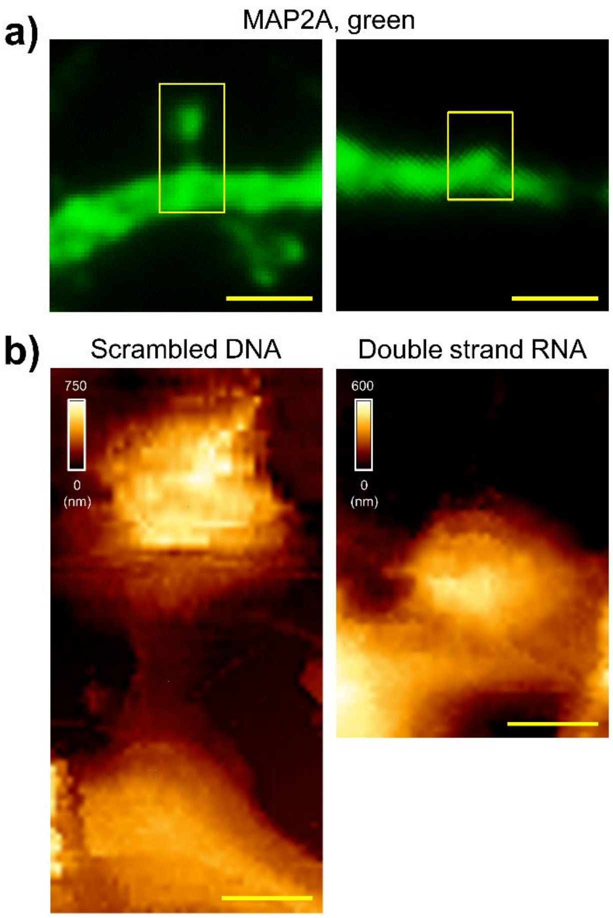

Figure S2. Control experiments to verify the specificity of the observed miR-132. a) Fluorescence images of mature spines (a mushroom type on the left and a stubby type in the right panel). Scale bars = 20 μm (left) and 5.0 μm (right). b) The topology of the spines in the boxed area (white dot line box in a) is obtained with AFM (3.0 × 6.0 μm or 3.0 × 4.0 μm). Scrambled DNA (22-nt) for miR-132 (left panel) and miR-132 complementary RNA is applied (right panel), and no cluster was observed in either condition. Scale bars = 1.0 μm.

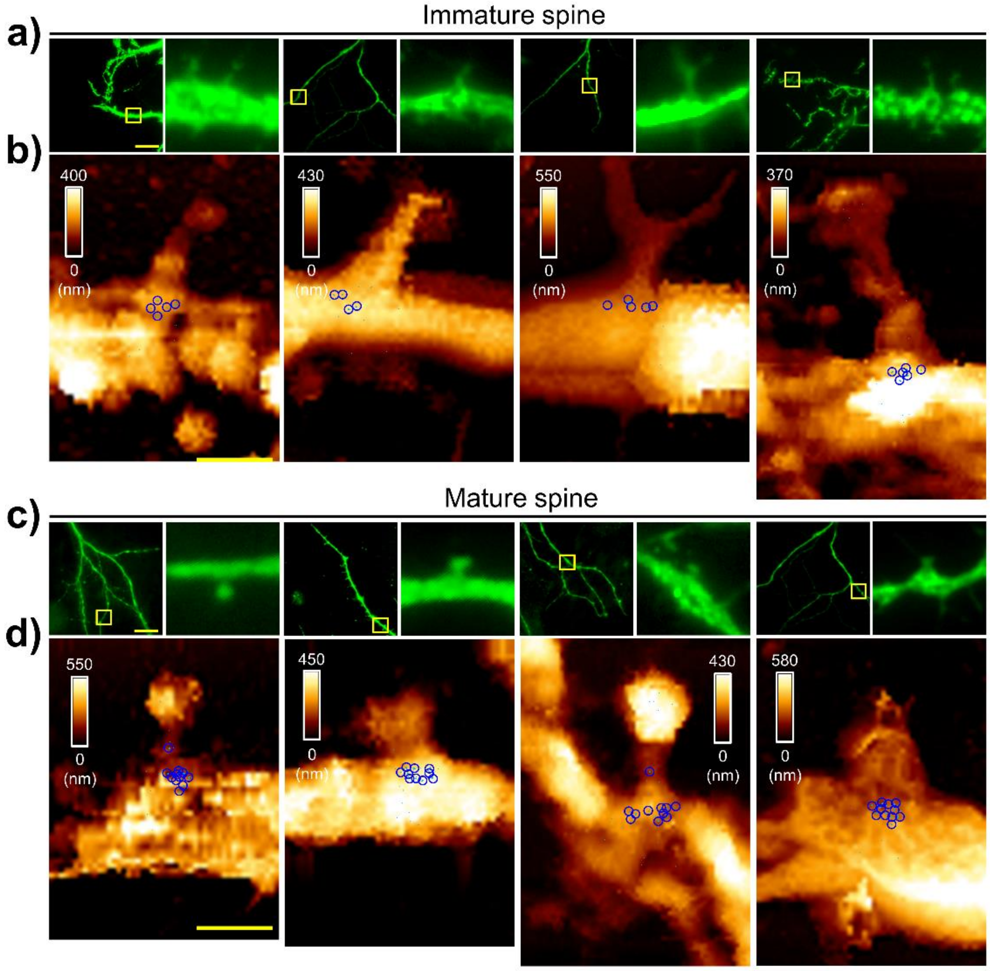

Figure S3. Visualization of individual miR-132 molecules next to various dendritic spines. a, c) Fluorescence images showing immature and mature spines (at DIV14). The right panels show the boxed areas in the left panels at a higher magnification. MAP2, green. b, d) AFM topographic images of the boxed areas in panels a) and c), with the force maps overlaid. Sky-blue pixels represent locations where specific unbinding events were observed in more than two of five measurements (pixel size, 10 nm), and blue circles indicate clusters corresponding to the hydrodynamic radius observed at high resolution. Scale bars: 20 µm (panel a and c, left image for each pair), 5.0 µm (panel a and c, right image for each pair), and 1.0 µm (panels in b and d).

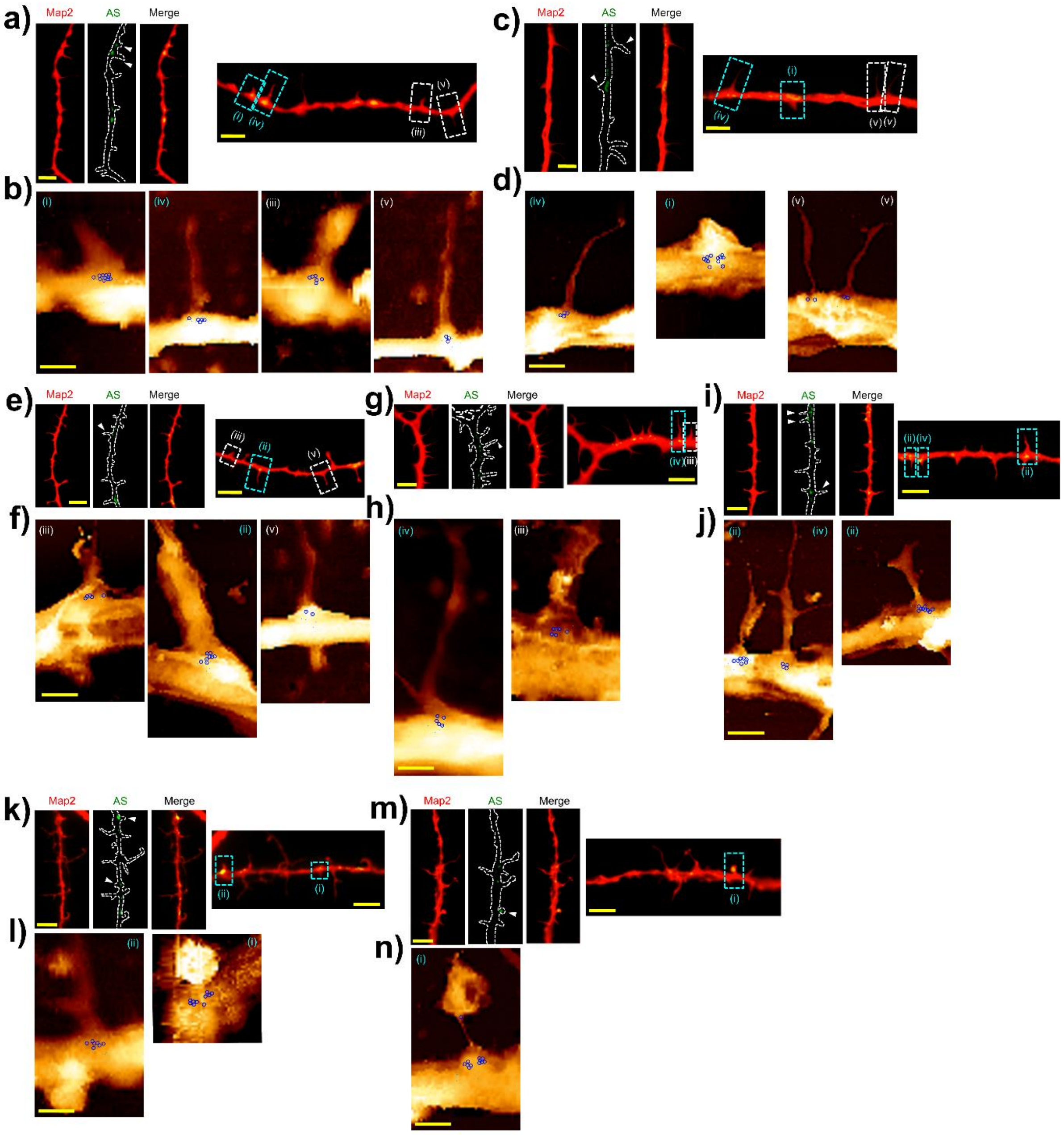

Figure S4. miR-132 mapping at active and inactive filopodia and dendritic spines labeled with ArcMin-AS. a, c, e, g, i, k, m) Fluorescence images of filopodia and dendritic spines labeled with ArcMin-AS (AS): active-mature spine (i), active-immature spine, (ii) and active-filopodium (iv) vs. inactive-immature spine (iii) and inactive-filopodium (v). b, d, f, h, j, l, n) AFM topographic images and force maps are obtained from the boxed areas of a, c, e, g, i, k, and m, respectively. Scale bars: 20 μm (a, c, e, g, i, k, m, left images in each set), 5.0 μm (a, c, e, g, i, k, m, right images in each set), and 1.0 μm (b, d, f, h, j, l, n).

	Observed number of miR-132s in a spine						
	Class	Spine 1	Spine 2	Spine 3	Spine 4	Spine 5	Mean ± SEM
Naïve	M	13	11	10	11	11	11.2 ± 0.49
	Im	5	6	5	4	5	5.00 ± 0.32
	Class	Spine 1	Spine 2	Spine 3	Spine 4	Spine 5	Mean ± SEM
ArcMin-AS (Active)	M	12	10	13	11	12	11.6 ± 0.51
	Im	8	7	7	9	8	7.80 ± 0.37
	F	4	5	4	5	4	4.40 ± 0.24
	Class	Spine 1	Spine 2	Spine 3	Spine 4	Spine 5	Mean ± SEM
ArcMin-AS (Inactive)	Im	6	5	5	6	5	5.40 ± 0.24
	F	2	2	3	2	2	2.20 ± 0.20

Table S1. Numbers of miR-132 molecules in naïve (control) spines, spontaneously active filopodia and spines (certified by labeling of ArcMin-AS), and inactive filopodia and dendritic spines. This table indicates the mean and standard error of the mean (s.e.m.) of the cluster numbers in each class of spines and filopodia. Five mature spines and five immature spines were obtained from individual naïve cells. Five ArcMin-AS-labeled (activated) spines, five unlabeled (inactivated) spines, and five filopodia were obtained from nine cells. M, mature spine; Im, immature spine; F, filopodium.

References

[1] I. Park, H. J. Kim, Y. Kim, H. S. Hwang, H. Kasai, J. H. Kim, J. W. Park, *Proc. Natl. Acad. Sci. USA* **2019**, *116*, 9616.

[2] A. Hayashi-Takagi, S. Yagishita, M. Nakamura, F. Shirai, Y. I. Wu, A. L. Loshbaugh, B. Kuhlman, K. M. Hahn, H. Kasai, *Nature* **2015**, *525*, 333.

[3] E. Burova, E. Ioffe, *Gene Ther.* **2005**, *12*, S5.
